# Capsular warning syndrome: clinical analysis and treatment

**DOI:** 10.1186/s12883-019-1522-0

**Published:** 2019-11-13

**Authors:** Lanying He, Ronghua Xu, Jian Wang, Lili Zhang, Lijuan Zhang, Fangfang Zhou, Weiwei Dong

**Affiliations:** 1 Department of Neurology, The Second People’s Hospital of Chengdu, Chengdu, 610021 People’s Republic of China; 2 Department of Neurosurgery, The Second People’s Hospital of Chengdu, Chengdu, 610021 People’s Republic of China; 3 Department of Neurology, The Second Affiliated Hospital of Chengdu College, Nuclear Industry 416 Hospital, Chengdu, 610021 People’s Republic of China; 4 Department of Neurology, First Affiliated Hospital, Chongqing Medical University, Chongqing, China, 400030 People’s Republic of China

**Keywords:** Capsular warning syndrome, Clinical characteristics, transient ischemic attack, Antiplatelet therapy, Thrombolysis

## Abstract

**Background:**

Capsular warning syndrome (CWS) is a rare clinical syndrome, which is defined as a recurrent transient lacunar syndrome. The mechanism and clinical management of CWS remain unclear. The aim of the study was to discuss the clinical characteristics of CWS and evaluate the different outcome between rt-PA and no rt-PA therapy.

**Methods:**

The present multicenter retrospective study involved three medical centers, and the clinical data were collected from patients with CWS between *January 2013* and December 2018. The clinical characteristics of CWS were analyzed. Patients were divided into two groups: rt-PA and no rt-PA groups. The therapeutic effects and prognosis of these two groups were analyzed. A good prognosis was defined as 3-month modified Rankin Scale (mRS) ≤ 2.

**Results:**

Our study included 72 patients, 27 patients were assigned to rt-PA group, 45 in no rt-PA group. Hypertension and dyslipidemia were the most common risk factors. The mean number of episodes before irreversible neurological impairment or the symptoms completely disappeared was five times (range: 3–11 times). A total of 58 (80.55%) patients had acute infarction lesions on the diffusion weighted imaging (DWI). The most common infarct location was the internal capsule (41,70.69%), followed by the thalamus and pons. The difference in therapeutic effects between the rt-PA, single and double antiplatelet groups was not statistically significant (*P* > 0.05). A good prognosis was observed in 61 (84.72%) patients after 3 months, in which 23 (23/27, 85.19%) patients were from the rt-PA group and 38 (38/45,84.44%) patients were from the no rt-PA group (*P* > 0.05). After 3 months of follow-up, two patients had recurrent ischemic stroke.

**Conclusion:**

The most effective treatment of CWS remains unclear. Intravenous thrombolysis is safe for CWS patients. Regardless of the high frequency of infarction in CWS patients, more than 80% patients had a favorable functional prognosis.

## Background

The term ‘Capsular Warning Syndrome’ (CWS) was first proposed in 1993 by Donnan et al. This was defined as having at least three stereotyped episodes of motor lacunar syndrome (MLS) or sensorimotor lacunar syndrome (SMLS) within 24 h, with complete recovery between episodes, which involved two of three body parts (face, arm, or leg) or more, without cortical symptoms [1]. Latter studies used a broader 72-h time range to define CWS. The other time intervals included 48 h or even 7 days [2, 3].

CWS is a rare clinical syndrome that presents as repeated stereotyped episodes of transient ischemic attacks (TIA), and increases the risk of permanent infarction. A previous study revealed that the incidence was approximately 1.5% among patients with TIAs [2]. Since the exact pathophysiological mechanism of CWS remains unclear, and different treatments have been suggested, such as blood pressure control [4, 5], anticoagulation [6], double antiplatelet therapy [7, 8], single antiplatelet and thrombolytic agent [8–14], the best clinical management of these patients remains controversial.

In the present study, patients with CWS were collected from three medical centers to discuss the clinical characteristics of CWS and evaluate the different outcome between rt-PA and no rt-PA therapy.

## Methods

### Patients and clinical data

CWS was defined as the succession of at least three episodes of stereotyped pure motor syndrome, pure sensory syndrome, sensory motor syndrome, ataxic syndrome, or clumsy hand, with or without dysarthria within a period 48 h, with a complete resolution between episodes.

Three authors (LY H, FF Z and LJ Z) carried out evaluation of all acute ischemic stroke (AIS) and TIA patients by searching the Electronic Medical Record (EMR) system of the three hospitals between *January* 2013 and December 2018. A data extraction form was designed and used to record the patient characteristics. Data included patients’ demographic information (including sex, age, current smoking, current alcohol drinking > 100 g/day, risk factors, medications use), infarct location and treatment.

The National Institutes of Health Stroke Scale (NIHSS) was used to assess the severity of stroke. The ABCD2 score was calculated at admission.

The 3-month modified Rankin Scale (mRS) and NIHSS score were obtained from the medical records or each primary physician, or by face-to-face interview. A good prognosis was defined as mRS ≤2.

### Statistical analysis

First, the clinical characteristics of all CWS patients were summarized. Continuous variables were expressed as median values (interquartile range [IQR]), numbers (%), or mean ± standard deviation (SD), and categorical variables were expressed as frequencies and percentage. Second, patients were classified into two groups: no rt-PA group and rt-PA group. Then, the demographic characteristics and prognosis were compared between these two groups in the univariate analysis. Continuous variables were compared using Student’s *t*-test. Categorical variables were compared using chi-square test or Fisher’s exact test. The distributions of continuous variables were determined using the Kolmogorov–Smirnov test, while Mann–Whitney two-sample test was applied in case of non-normal distributions. The data was analyzed using the SPSS 22.0 software. *P* < 0.05 was considered statistically significant.

## Results

### Demographics characteristics of the study subjects

**Fig. 1:**
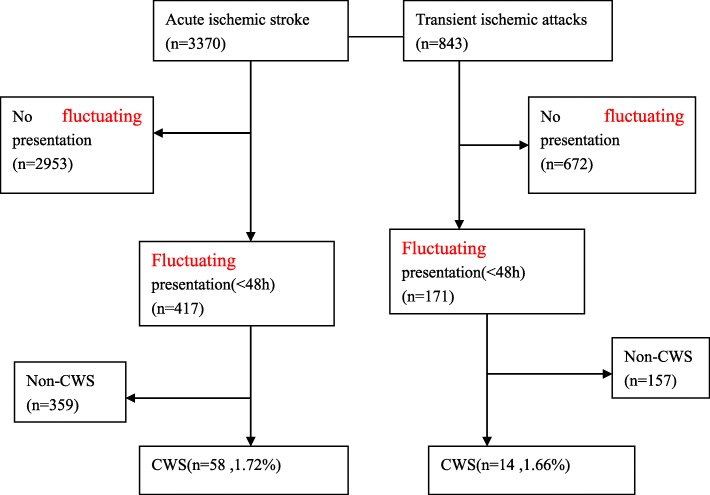
Patient’s flowchart

The EMRs of 4213 patients were retrospectively reviewed. Among these patients, 3370 patients had AIS and 843 patients had TIA. The EMRs were manually reviewed to identify patients affected by CWS. After reviewing the patient’s history, clinical characteristics, risk factors and neuroimaging, it was found that 417 AIS patients had fluctuating presentations, while 171 TIA patients had fluctuating presentations (Fig. 1). After the manual review, 72 patients with CWS were identified (72/4213, 1.71%), which comprised of 42 (58.30%) male patients and 30 (42.70%) female patients. The mean age of these patients was 66.28 ± 7.90 years old (range: 38–96 years old).

In the study population, the most common risk factors were hypertension and dyslipidemia. Among these patients, 52 patients had a history of hypertension, 47 had a history of hyperlipidemia, 27 had diabetes, 26 patients were currently smokers, and 24 patients were currently alcohol drinkers(> 100 g/day). The clinical characteristics of CWS patients are presented in Table 1.

**Table 1:** Clinical characteristics of patients with CWS

	*N* = 72
Age, year (Mean ± SD);	66.28 ± 7.90;
NIHSS score, median (IQR)	8 (5–9)
Systolic pressure, mmHg (Mean ± SD)	167.75 ± 22.76
Glucose, mmol/l (Mean ± SD)	6.58 ± 1.81
Mean number of episodes, n (Mean ± SD)	5.31 ± 2.22
Mean Duration, min (Mean ± SD)	24.11 ± 11.21
ABCD2 score, median (IQR)	5 (4–5)
Men, n (%)	42 (58.33)
BMI ≥ 24 kg/m, n (%)	16 (22.22)
Hypertension, n (%)	52 (72.22)
Current Smoking, n (%)	26 (36.11)
Current alcohol drinking, n (%)	24 (33.33)
Diabetes, n (%)	27 (37.50)
Hyperlipidemia, n (%)	47 (65.28)
Family history of stroke, n (%)	16 (22.22)
Medications use	
Antiplatelet, n (%)	22 (30.56)
Antihypertensive, n (%)	31 (43.06)
Lipid-lowering medications, n (%)	36 (50.00)
Lacunar syndrome	
Pure motor syndrome, n (%)	32 (44.44)
Pure sensory syndrome, n (%)	12 (16.67)
Sensory motor syndrome, n (%)	24 (33.33)
Ataxic hemiparesis, n (%)	4 (5.56)
Infarct location (58)	
Internal capsule, n (%)	41 (70.69)
Pons, n (%)	5 (8.62)
Striatum, n (%)	2 (3.44)
Thalamus, n (%)	7 (12.07)
Midbrain, n (%)	3 (5.17)
Treatment	
Rt-PA, n (%)	27 (37.50)
Double Antiplatelet, n (%)	30 (41.67)
Single Antiplatelet (15)	
Aspirin, n (%)	12 (16.67)
Clopidogrel, n (%)	3 (4.17)

### Clinical characteristics

The median NIHSS score at admission was 8 (range: 2–12). A total of 59 patients (81.94%) had elevated blood pressure when they were admitted to the stroke ward, and their mean systolic pressure was 167.75 mmHg. Furthermore, 32 (44.44%) patients had pure motor syndrome, 24 (33.33%) patients had sensory motor syndrome, 12 (16.67%) patients had pure sensory syndrome, and 4 (5.56%) patients had ataxic syndrome. The mean number of episodes before irreversible neurological impairment or the symptoms completely disappeared was five times (range: 3–11). The mean duration of episodes was 24 min (range: 2–50). After CWS, permanent neurological impairment occurred in 52 (72.22%) patients, and the symptoms completely disappeared in 20 patients (27.78%).

### Complementary examinations

A total of 28 (65.28%) patients had hyperlipidemia on admission. The carotid artery ultrasonography revealed that 41 (56.94%) patients had mild carotid artery plaque. All patients were subjected to brain computed tomography (CT) and 24-h electrocardiogram (ECG) monitoring on admission. No arrhythmia was found during the ECG monitoring, and the echocardiography did not reveal any cardiac embolic sources in these patients. The cranial CT revealed no acute cerebral infarctions on admission. The intracranial and extracranial vasculature were evaluated through computed tomography angiography (CTA) in all patients, which was conducted within 5 days after admission. The cranial CTA revealed no severe stenosis or dissection in all patients.

A brain magnetic resonance imaging (MRI) was performed at 12–48 h after admission for all patients, and a total of 58 patients had an acute infarction on the diffusion-weighted imaging (DWI). All the ischemic lesions found in the MRI were subcortical, the size of the lesion was < 15 mm, and the most frequent infarct area was the internal capsule (41, 70.69%), while other locations were in the thalamus (7, 12.07%), pons (5, 8.62%), midbrain (3, 5.17%), and striatum (2, 3.44%).

### Treatment

A total of 27 (37.50%) patients were treated with rt-PA on admission, these patients were treated with antiplatelet therapy (aspirin or clopidogrel) after 24 h, when hemorrhage was excluded by the CT examination. A total of 45 patients were treated with antiplatelet therapy, among these patients, 30 patients were treated with 300 mg of clopidogrel, followed by 75 mg/day of the same, and aspirin (100 mg/day) plus high-dose atorvastatin (40 mg/day), 12 patients were treated with 200 mg of aspirin plus high-dose atorvastatin, and three patients were treated with clopidogrel plus high-dose atorvastatin on admission. The baseline characteristics of patients in the rt-PA group and no rt-PA group were compared (Table 2). Age, NIHSS score and the mean number of episodes were higher in the no rt-PA group than in the rt-PA group, but the difference was not statistically significant (*P* > 0.05). There were no significant group differences in the percentage of hypertension, diabetes and hyperlipidemia (*P* > 0.05).

**Table 2:** Comparison of baseline characteristics between patients in the rt-PA and no rt-PA groups

	rt-PA group (27)	no rt-PA group (45)	P*
Age, year, (mean SD)	64.69 ± 7.11	67.24 ± 8.27	0.179
NIHSS score at onset, median (IQR)	7 (4–8)	8 (6–9)	0.088
Mean number of episodes, n (%)	4.74 ± 2.31	5.76 ± 2.44	0.142
Mean duration, minutes (mean SD)	24.4 ± 11.64	23.91 ± 11.08	0.926
ABCD2 score, median (IQR)	5 (4–5)	5 (4–5)	0.967
Systolic pressure, mmHg (mean SD)	165.15 ± 19.00	169.31 ± 24.81	0.340
Glucose, mmol/l (mean SD)	5.72 ± 0.99	6.10 ± 1.10	0.124
Men, n (%)	18 (66.67)	26 (57.78)	0.789
BMI ≥ 24 kg/m, n (%)	7 (25.93)	9 (20.00)	0.558
Hypertension, n (%)	20 (74.07)	32 (71.11)	0.786
Currently smokers, n (%)	8 (29.63)	18 (40.00)	0.375
Diabetes, n (%)	11 (40.74)	16 (35.57)	0.660
Hyperlipidemia, n (%)	17 (62.96)	30 (66.67)	0.749
Currently alcohol drinkers, n (%)	9 (33.33	15 (33.33)	1.00
Family history of stroke, n (%)	7 (25.93)	9 (20.00)	0.558
Medications use			
Antiplatelet, n (%)	8 (29.63)	14 (31.11)	0.895
Antihypertensive, n (%)	12 (44.44)	19 (42.22)	0.854
Lipid-lowering medications, n (%)	11 (40.74)	25 (55.56)	0.224

*The comparison between the rt-PA and no rt-PA groups. Continuous variables were expressed as mean ± standard deviation (SD). Categorical variables were expressed as frequencies and percentages. Continuous variables were compared using Student *t*-test. Categorical variables were compared using chi-square test or Fisher’s exact test. The distributions of continuous variables were determined using the Kolmogorov–Smirnov test, while Mann–Whitney two-sample test was applied in case of non-normal distributions

No bleeding complications occurred in the rt-PA group. A total of 17 patients presented new episodes after rt-PA treatment, 22 patients had ischemic infarction, and 10 patients had a complete recovery. Patients in the no rt-PA group received antiplatelet therapy, in which 35 patients presented with new episodes (9 patients received aspirin, 2 patients received clopidogrel, and 24 patients received double antiplatelet). Furthermore, 10 patients in the no rt-PA group had a complete recovery (3 patients received aspirin, 1 patient received clopidogrel, and 6 patients received double antiplatelet). Moreover, 36 patients in the no rt-PA group had acute infarctions in the DWI (9 patients received aspirin, 3 patients received clopidogrel, and 24 patients received double antiplatelet). The patients who receive rt-PA therapy appeared to have a higher percentage of complete recovery, but the difference in therapeutic effects among the rt-PA, single and double antiplatelet groups were not statistically significant (*P* > 0.05, Table 3).

**Table 3:** Comparison of therapeutic effects among rt-PA, single and double antiplatelet therapy

	Single antiplatelet group (15)	Double antiplatelet group(30)	rt-PA group (27)	P*
New episodes after treatment, n (%)	11 (73.33)	24 (80.00)	17 (62.96)	0.356
Complete recovery, n (%)	4 (26.67)	6 (20.00)	10 (37.04)	0.356
Acute infarction, n (%)	12 (80.00)	24 (80.00)	22 (81.48)	0.998

*The comparison among rt-PA, single and double antiplatelet therapy. Categorical variables were expressed as frequencies and percentages. Categorical variables were compared using chi-square test or Fisher’s exact test

A total of 61 patients (84.72%) had a good outcome after 3 months. Among these patients, 23 patients were from the rt-PA group, and 38 patients were from the no rt-PA group (26 patients received double antiplatelet, 10 patients received aspirin, and 2 patients received clopidogrel). Furthermore, 2 patients had recurrent ischemic stroke (1 patient received aspirin, and 1 patient received clopidogrel). Compared with the baseline, the NIHSS score after 3 months decreased in both two groups. There was no significant difference in the 3-month prognosis between the two groups (*P* > 0.05, Table 4).

**Table 4:** The prognosis of CWS patients in the rt-PA and no rt-PA groups at 3 months

	rt-PA group (27)	no rt-PA group (45)	P*
mRS ≤ 2, n (%)	23 (85.19)	38 (84.44)	0.933
NIHSS score	3.26 ± 2.05	3.49 ± 1.84	0.682

*The comparison between the rt-PA and no rt-PA groups. Continuous variables were compared using Student’s *t*-test. Categorical variables were compared using chi-square test

## Discussion

CWS is a rare clinical syndrome that has not been extensively studied. Previous studies have shown that the incidence of CWS in TIA patients was 1.5–4.5% [1–3]. In the present study, patients with recurrent lacunar syndromes up to 48 h from the first episode were included. The percentage in AIS/TIA was 1.71%, which appears to be consistent with that in previous studies.

CWS has been characterized by the abrupt onset of symptoms, and the duration of each episode varies. One study revealed that the mean duration of each episode was 6.1 min [1]. In the present study, for patients with recurrent TIA presentations within several hours from the first episode, the symptoms completely improved within 2–50 min, and the mean duration of each episode was 24 min. CWS has generally manifested as a repetitive lacunar syndrome, although some authors have reported that CWS might be associated with other symptoms, such as sensory dullness and ophthalmoplegia [15]. The most frequent symptom was MLS. In a previous study, Donnan et al. recruited 50 patients with CWS, and the percentage of MLS was 50% [1]. In another study, Camps-Renom et al. reported that MLS accounted for 61.9% of 42 CWS cases [16]. In the present study, 32 patients mainly manifested with pure motor syndrome, while 24 patients manifested with sensory motor syndrome, and the frequency of pure motor syndrome/sensory motor syndrome was 77.78%. This present finding was consistent with a recent study [17].

CWS has a high risk of developing ischemic stroke with a permanent deficit. The 7-day stroke risk following CWS can reach as high as 60, 71.2% patients had acute ischemic lesions with the routine use of MRI [2]. The most frequent location was in the internal capsule (50%), some studies have revealed that ischemic lesions can occur at other locations [16, 18]. In our study, 58 (80.56%) patients had an acute infarction on DWI, the most frequent location was in the internal capsule, which occurred in 41 (70.69%) patients, while the other locations were in the thalamus, midbrain, pons and striatum, the results were consistent with that in previous studies [16, 18].

The exact pathophysiological mechanism of CWS remains unclear. Studies have confirmed that hypertension, diabetes, dyslipidemia, smoking and other common stroke risk factors are correlated to CWS, which might suggest that atherosclerosis may be involved in the pathogenesis of CWS [1, 19–21]. Some authors have speculated that CWS was most likely to be ischemia due to in situ small-penetrating vessel disease, while some authors have considered that intermittent hemodynamic changes secondary to structural arterial changes or hypertension might be the most likely mechanism of CWS [1, 22]. In the present study, the cranial CTA revealed that vascular stenosis or dissection was not found in patients, while echocardiography did not reveal any cardiac sources. Therefore, we suspect that the pathogenesis of CWS may be correlated to the arteriosclerosis of small-penetrating vessels. However, the mechanism of the symptom fluctuations remains unclear.

There has been some debate on the most effective treatment in the acute phase of CWS. Regardless of the various available treatments being used, it remains unclear whether these treatments alter the natural course of the syndrome. Intravenous thrombolysis, double antiplatelet therapy, single antiplatelet therapy, anticoagulants, and vasopressors have been used to treat patients with CWS [1, 4, 5, 7, 9–13, 23–29]. However, it remains uncertain whether any of these therapies can change the progression of the syndrome. There was no strong evidence for the efficacy of anticoagulant therapy in the acute phase of CWS. Furthermore, a case series suggested that double antiplatelet therapy (aspirin with the addition of clopidogrel) might be beneficial, which might be similar to the effect observed in acute coronary syndromes [7, 28, 29]. In a case series that includes two patients with CWS, it was reported that following the start of double antiplatelets, there was no progression of symptoms [8]. A retrospective study revealed that double antiplatelet therapy could decrease clinical fluctuations and improve functional outcome [30]. However, there were also reports that double antiplatelet therapy cannot prevent infarction. In a recent study, 17 patients with stuttering lacunar syndrome (SLS) were treated with double antiplatelet therapy. A loading dose of 300 mg of clopidogrel was administered along with aspirin in all cases, and the symptoms improved in 11 patients. Hence, the authors reported that double antiplatelet therapy appears to be effective for the acute treatment of SLS [17]. In the present study, 25 patients suffered from infarction, even though they received double antiplatelet therapy, while 12 patients treated with single antiplatelet therapy had infarction, and 2 patients treated with single antiplatelet therapy had recurrent ischemic stroke within 3 months. The result showed that double antiplatelet therapy may be more effective than aspirin or clopidogrel alone, as a secondary prevention strategy in CWS.

There is little evidence on whether thrombolysis is beneficial for CWS. Some studies have shown that for CWS patients treated with thrombolysis, the symptoms completely disappeared. Hence, some scholars have considered that rt-PA could be effective [31, 32]. However, a previous study revealed that there was no significant benefit in functional outcomes in nine patients who received intravenous thrombolysis, when compared to nine patients who did not received thrombolysis [3]. In a recent study, it was revealed that intravenous thrombolysis could stop stuttering in patients with SLS. Therefore, the author considered that thrombolysis should be the therapeutic choice when patients with SLS present within the therapeutic time window with disabling symptoms [17]. In the present study, 27 patients were treated with intravenous rt-PA and 45 patients were treated with no rt-PA, the difference in therapeutic effects among the rt-PA, single and double antiplatelet groups was not statistically significant (Table 3). This might be correlated to the small sample size of the study. In the future, multicenter clinical randomized controlled trials should be conducted to explore the effect of thrombolytic therapy in CWS.

Finally, one of the aims of the present study was to analyze the prognosis between the rt-PA and no rt-PA groups. In the present study, a favorable outcome was observed in 61 (84.72%) patients after 3 months. Among these patients, 23 patients (23/27,85.19%) were from the rt-PA group and 38 (38/45,84.44%) patients were from the no rt-PA group. There was no difference in functional outcome at 3 months between the rt-PA and no rt-PA groups. In general, intravenous thrombolysis is safe for CWS patients, and no bleeding complications have been reported. Therefore, intravenous thrombolysis is an alternative treatment for CWS within 4.5 h after the onset of symptoms. Due to the small number of patients, the effect of rt-PA in CWS must be further confirmed in clinical studies.

Some limitations of the present study merit consideration. The present study updates some information about CWS. Since the present study was a multicenter and retrospective study, CWS is a rare clinical disease, and the number of included patients was small, these may have affected the results of the present study. A total of seven patients were followed up by face-to-face interview. The recollection of these patients on the functional outcome after 3 months of onset might lead to some recollection bias. In the future, there may be a need to further expand the number of patients and further explore the mechanism of CWS, in order to find the best treatment.

## Conclusion

In conclusion, CWS is a rare clinical syndrome, which describes recurrent stereotyped lacunar transient ischemic attacks clustered within a short period of time and is associated with a high risk of developing a completed stroke. The mechanism of CWS had not been fully elucidated. Various treatments are available and being used for CWS. However, the optimal treatment remains unclear. Intravenous thrombolysis is an alternative treatment for CWS within 4.5 h after the onset of symptoms. Regardless of the high incidence of infarction in CWS patients, most patients had a good prognosis after 3 months.

## Data Availability

The datasets used and/or analyzed during the current study are available from the corresponding author on reasonable request.

## Abbreviations

CTA: computed tomography angiography
CWS: Capsular warning syndrome
mRS: modified Rankin Scale
NIHSS: National Institutes of Health Stroke Scale
SD: Standard Deviation
SLS: Stuttering lacunar syndrome
